# Portable NIV for patients with moderate to severe COPD: two randomized crossover trials

**DOI:** 10.1186/s12931-021-01710-2

**Published:** 2021-04-26

**Authors:** Daniel Sebastian Majorski, Friederike Sophie Magnet, Saadia Thilemann, Claudia Schmoor, Wolfram Windisch, Sarah Bettina Schwarz

**Affiliations:** 1 Department of Pneumology, Cologne Merheim Hospital, Kliniken der Stadt Köln gGmbH, Witten/Herdecke University, Faculty of Health/School of Medicine, Cologne, Germany; 2 Clinical Trials Unit, Medical Center – University of Freiburg, Faculty of Medicine, University of Freiburg, Freiburg, Germany

**Keywords:** COPD, Respiratory insufficiency, Endurance capacity, Non-invasive ventilation

## Abstract

**Background:**

Long-term non-invasive ventilation (NIV) is as an established treatment option for chronic hypercapnic COPD patients. Beneficial effects have also been shown during exercise, but this is restricted to rehabilitation programs. New portable NIV (pNIV) devices may now enable NIV application during walking at home.

**Study design and methods:**

In two randomized crossover trials, the impact of pNIV on dyspnea and endurance capacity was investigated in patients with moderate to severe COPD. Participants performed a standardized 6-min walking test, with and without pNIV, using a pre-set inspiratory/expiratory positive airway pressure of 18/8 cmH_2_O. The first study was performed in NIV-naïve patients (Study I), while the second study was performed in those already established on long-term NIV (Study II).

**Results:**

38 patients (66.9 ± 7.4 years, mean FEV_1_: 30.3 ± 8%pred) and 23 patients (67.6 ± 8.7 years, mean FEV_1_: 29.8 ± 10.4%pred) participated in Study I and II, respectively. In Study I, the mean difference in the Borg Dyspnea Scale (BDS, primary outcome) score following walking was 3.2 (IQR 2–4) without pNIV, compared to 2.6 (IQR 1–4) with pNIV (ΔBDS 0.65, *P* = 0.04), while walking distance increased from 311.8 m (95%CI 276.9–346.6 m) to 326.3 m (95%CI 291.5–361.2 m) (*P* = 0.044) when pNIV was used. Accordingly, in Study II, the mean difference in BDS was 4.4 (IQR 3–6) without pNIV, compared to 4.5 (IQR 3–6) with pNIV (ΔBDS 0.09, *P* = 0.54), while walking distance decreased from 291.5 m (95%CI 246.1–336.9 m) to 258.4 m (95%CI 213–303.8 m) (*P* ≤ 0.001).

**Interpretation:**

The use of a pNIV device during walking can improve dyspnea and walking distance in patients with moderate to severe COPD. Patients who do not already receive long-term NIV therapy are more likely to benefit compared to those undergoing long-term NIV. Careful patient selection is mandatory.

*Clinical Trial Register*: DRKS00013203; DRKS00012913 registered October 20th 2017 and October 16th 2017; https://www.drks.de/drks_web/

## Background

The delivery of non-invasive ventilation (NIV) via a facemask serves as an established treatment option for patients with chronic hypercapnic respiratory failure, which can arise from a number of different etiologies including COPD [1–3]. For this purpose, NIV is typically used intermittently in a nocturnal setting—an approach that is capable of improving long-term survival and health-related quality of life [1]. In COPD patients, the use of NIV with ventilator settings that are high enough to normalize/improve hypercapnia has been established as the treatment modality of choice [4, 5].

In addition, NIV can also be used during physical activity, particularly in patients with COPD [6–8]. This serves to improve the outcome of rehabilitation therapy [9], and can also be used during walking in the home environment to improve exercise capacity and dyspnea. Accordingly, several studies carried out in various clinical settings have clearly shown that NIV-aided walking improves dyspnea, walking distance and gas exchange [10–14].

However, one major problem in transferring this experimentally positive result to the home environment is the fact that applying NIV techniques during walking can be laborious and cumbersome, since this form of therapy was originally developed for use during rest. For example, the use of a rollator [12] or backpack [15] to carry the NIV device is suggested to restrict the patient's range of motion and is therefore detrimental to its application in the home environment.

In order to overcome the burden of heavy ventilators, portable NIV devices (pNIV) have been developed. These products are generally used as hand-held devices, which can be carried in one hand. Thereby, most of the restrictions caused by the heavy home-NIV devices can be easily overcome. Of note, a recent pilot study of COPD patients was able to demonstrate that the use of pNIV with a preset inspiratory positive airway pressure (IPAP) of 18 cmH_2_O and a preset expiratory positive airway pressure (EPAP) of 8 cmH_2_O is capable of reducing the recovery time of dyspnea after exercise [16]. Therefore, this study showed that pNIV has the potential to improve exercise capacity.

However, it remains unclear exactly how pNIV improves exercise performance. In addition, the patient groups that would benefit most from pNIV use in terms of disease severity need to be identified. Therefore, the present study aimed to test the hypothesis that pNIV is capable of improving both dyspnea and walking distance in patients with COPD. It was further hypothesized that the success of pNIV is dependent on disease severity. To address these questions, two different randomized cross-over trials were performed in COPD patients with moderate to severe airflow limitation using a comparable study design. Patients without established long-term NIV formed the cohort for the first study, while long-term NIV patients formed the cohort for the second study.

## Material and methods

Two crossover randomized trials with different study populations but an identical study design were performed at the Department of Pneumology, Lung Clinic, Cologne Merheim Hospital, Witten/Herdecke University, Germany. Both study protocols were approved by the ethics committee at Witten/Herdecke University. The studies were performed in accordance with the ethical standards laid down in the Declaration of Helsinki [17] and were separately registered at the German Clinical Trials Registry (DRKS00012913: Study I; DRKS00013203: Study II). All patients provided written informed consent.

### Patients

COPD patients were included if they presented a moderate to severe airflow limitation which, according to the Global Initiative for Obstructive Lung Disease (GOLD), was defined by a forced expiratory volume in one second (FEV_1_) < 50% predicted [18]. The first study (Study I) consisted of COPD patients (COPD stages GOLD III and IV) without chronic hypercapnic respiratory failure (DRKS00012913). The second study (Study II, DRKS00013203) consisted of patients with established long-term NIV aimed at treating hypercapnic respiratory failure, as per German medical guidelines [2, 3]. Only patients receiving long-term NIV for more than 3 months were included in Study II. In contrast, being on long-term NIV for any time served as a strict exclusion criterion for study I. In addition, patients with CPAP therapy were excluded in both studies. At the time of recruitment, patients were undergoing optimal inhalative therapy [19] and no subsequent changes to medication were made during the trial. All patients were required to be free of exacerbation—which was defined by any medical treatment aggravation including antibiotics and systemic corticosteroids—at least 4 weeks prior to study inclusion [19]. In addition, patients with any type of unsteadiness or advancing difficulties in walking were excluded.

### Study design and measurements

Prior to exercise testing, patients underwent a familiarization session for the pNIV, which included detailed instructions on how to handle the device as well as a practice session lasting at least 30 min. For exercise testing, a 6-min walking test (6MWT) was performed according to previous recommendations [20]. When necessary, long-term oxygen therapy (LTOT) was used during walking using flow rates that were prescribed prior to study enrollment. Blood gas analysis (ABL 90, Radiometer GmbH, Willich, Germany), spirometry, full body plethysmography, and respiratory muscular function testing was performed (ZAN500 Bodyplethysmograph, ZAN Austria, Winkling, Austria). For both crossover trials, patients were randomly assigned to exercise with and without the pNIV device using two cross-over sequences:."Period 1: 6MWT with pNIV/Period 2: 6MWT without pNIV" and "Period 1: 6MWT without pNIV/Period 2: 6MWT with pNIV". Patients were free to decide when and how often they wanted use the pNIV device during their walking sessions.

### Portable non-invasive ventilation (pNIV)

**Fig. 1:**
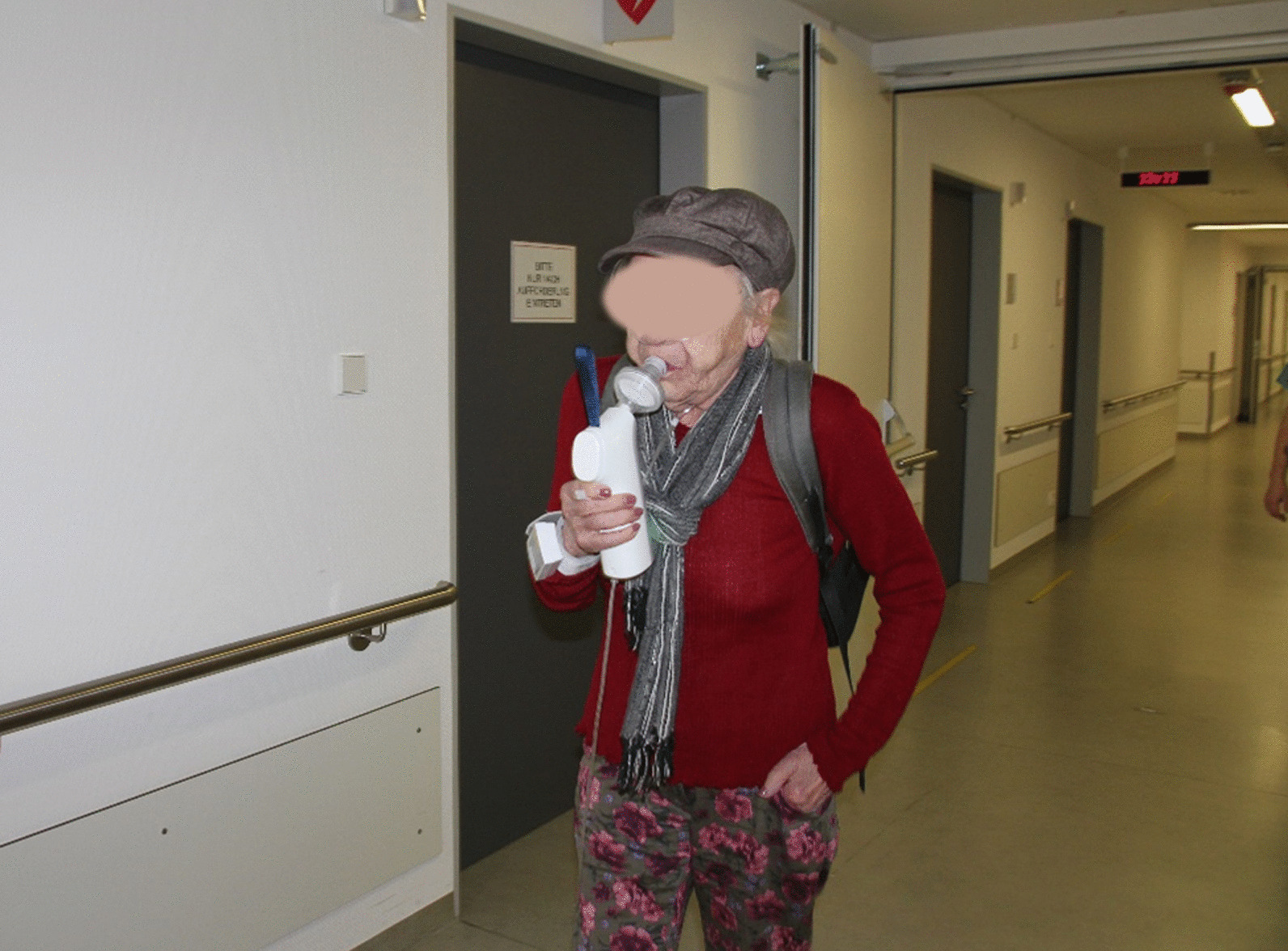
A patient using the pNIV device while completing a 6-min walking test

The certified medical device 'VitaBreath' (Philips, Respironics, Murrysville, PA, USA) was used as the pNIV device for the purpose of both studies. It is a hand–held device specifically designed to provide COPD patients with positive pressure via a mouth piece, during or after exercise, in order to relieve breathlessness caused by physical exertion (Fig. 1) [21]. The pNIV device provides two fixed pressure levels: 18 cmH_2_O during inspiration and 8 cmH_2_O during expiration.

### Statistical analysis

The primary endpoint was the difference in the Borg Dyspnea Scale (BDS) score before and after exercise. This decision was based on the observation that exercise in these patients is severely restricted by symptoms and also on the acceptation that using external devices during physical activity in clinical practice will only occur in case of patients subjectively benefitting from the intervention. In each study, the hypothesis that there is no difference in dyspnea between interventions was tested at two-sided level alpha = 0.05 against the alternative hypothesis that there is a difference.

For sample size calculation based on the BDS score, a standard deviation of 3 points was assumed, and the difference between interventions was assumed as 1.5 points in study I and as 2 points in study II. This resulted in a required sample size of 38 patients in study I and of 22 patients in study II to achieve a power of 80%. In the analysis, exact 2-sample Wilcoxon tests of crossover differences at two-sided level alpha = 0.05 were used. In addition, tests of period and of carryover effects (i.e. an interaction between intervention and period) were performed using exact Wilcoxon tests.

Walking distance, blood gas analysis, blood pressure and heart rate served as secondary outcomes. The effects of the intervention on these secondary endpoints were evaluated using linear regression models. The factors intervention, period and randomized sequence were included as fixed effects, and the patient within the randomized sequence was included as a random effect. The effects of the intervention were estimated with 95% confidence intervals and tested at the two-sided level alpha = 0.05. Furthermore, tests for a period effect and carryover effects were performed in these models. No alpha adjustment was performed for the multiple testing of the secondary endpoints. The p-values of the statistical tests of effects on the secondary endpoints should be interpreted in the context of a descriptive analysis.

## Results

**Fig. 2:**
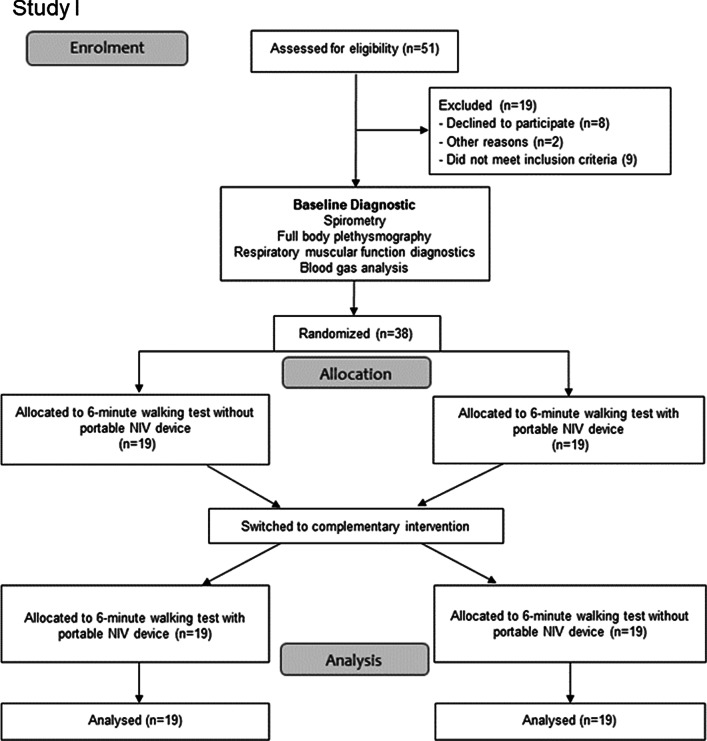

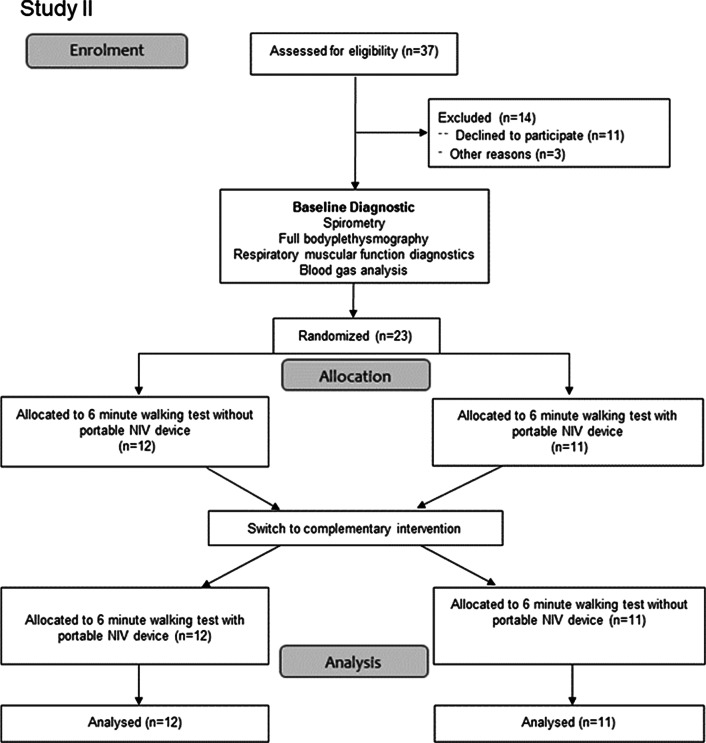
**a** CONSORT flow diagram of the study enrolment process (Study I) [27]. **b** CONSORT flow diagram of the study enrolment process (Study II) [27]. The discrepancy between the sample size calculation and the final recruitment size is due to the recruitment process. There was no post-recruitment

Out of the 94 patients with severe COPD who were screened, 61 patients met the inclusion criteria and were consecutively enrolled (Fig. 2a and b). Demographic data, blood gases, data on bodyplethysmography and respiratory muscle function testing are presented in Table 1. The ventilator settings used for established long-term NIV therapy were as follows: an IPAP level of 23.7 ± 3.8 cmH_2_O (IQR 20.25–26.75) and an EPAP level of 5.5 ± 1 cmH_2_O (IQR 5–6). The results of the blood gas analyses, pulseoximetry and blood pressure measurement for each of the exercise testing sequences (with or without the pNIV device) are illustrated in Tables 2 and 3.

**Table 1:** Patient characteristics at baseline in Studies I and II

	Total (N = 61)	Study I* (N = 38)	Study II^#^ (N = 23)	*P*
Age [years]	67 ± 8	66.9 ± 7.4	67.6 ± 8.7	0.74
Female gender [%]	37.7	32	47.8	0.25
FEV_1_ [%pred]	30.3 ± 8.9	30.3 ± 8	29.8 ± 10.4	0.99
FEV_1_ [liter]	0.85 ± 0.26	0.88 ± 0.26	0.78 ± 0.25	0.25
FVC [%pred]	66.25 ± 18.9	63.2 ± 19.9	70.8 ± 15.7	0.13
FVC [liter]	2.37 ± 0.86	2.4 ± 0.9	2.31 ± 0.81	0,67
FEV_1_/FVC ratio [%]	37 ± 10.4	37.5 ± 10.3	35.7 ± 10.4	0.31
TLC [%pred]	117.6 ± 20.9	117.7 ± 18.8	117.3 ± 23.9	0.94
RV [%pred]	202.5 ± 54.7	203.7 ± 49.6	200.5 ± 68.5	0.84
P0.1 [kPa]	0.48 ± 0.25	0.53 ± 0.25	0.42 ± 0.14	0.25
PImax [kPa]	5.5 ± 22.3	5.9 ± 2.3	4.9 ± 2	0.16
PEmax [kPa]	10.0 ± 3.9	9.6 ± 3.7	10.7 ± 4.1	0.41
Nasal sniff [kPa]	4.8 ± 1.5	5.4 ± 1.5	4.1 ± 1.6	0.01
Cumulative smoking dosage [pack years]^Υ^	53.1 ± 23.6	55.2 ± 23.4	49.8 ± 23.4	0.32
BMI [kg/m^2^]	26.6 ± 5.9	24.5 ± 3.9	30 ± 6.9	0.003
pH	7.42 ± 0.03	7.42 ± 0.03	7.41 ± 0.03	0.4
PaO_2_ [mmHg]	65.8 ± 11.7	60.6 ± 6.8	74.3 ± 13	< 0.001
PaCO_2_ [mmHg]	42.4 ± 6.1	41.3 ± 6.9	44.4 ± 3.6	0.01
HCO_3_^−^ [mmol/l]	27 ± 2.4	26.3 ± 2.2	28.2 ± 2.3	0.01
Supplemental oxygen [% of patients]; [l/min]**	38.3; 2.6 ± 1.2	18.4; 1.9 ± 0.6	76; 3 ± 1.3	< 0.001

*FEV*_*1*_ forced expiratory volume in one second, *FVC* forced vital capacity, *RV* residual volume, *TLC* total lung capacity, *BMI* body mass index, *PaO*_*2*_ partial pressure of arterialized oxygen, *PaCO*_*2*_ partial pressure of arterialized carbon dioxide, *HCO*_*3*_^*−*^ arterialized standard hydrogen carbonate

*Patients without long-term NIV; **Only O_2_ dependent patients

^#^Patients with long-term NIV

^Υ^40 patients were ex-smokers, 21 patients were active smokers;

**Table 2:** Study I*: blood gas analyses and vital parameters after exercise (N = 38)

Study I*	Period	With pNIV	Without pNIV	Treatment effect (95% CI)	*P*
pH	1	7.42 ± 0.04	7.4 ± 0.05	− 0.005 [− 0.01; 0.004]	0.26
	2	7.39 ± 0.05	7.42 ± 0.04		
PaCO_2_ [mmHg]	1	42.7 ± 9.1	41.4 ± 6.1	0.46 [− 0.8; 1.7]	0.48
	2	42.3 ± 5.9	43.2 ± 8.4		
PaO_2_ [mmHg]	1	66.7 ± 7.4	61.1 ± 8.9	0.03 [− 1.9; 2]	0.98
	2	58.5 ± 8.4	63.4 ± 8.3		
HCO_3_^−^ [mmol/l]	1	26.4 ± 2.4	24.8 ± 2	− 0.2 [− 0.7; 0.3]	0.45
	2	24.7 ± 2.7	26.6 ± 2.3		
S_a_O_2_ [%]	1	93.6 ± 2	90.8 ± 5.3	− 0.2 [− 1.4; 0.8]	0.63
	2	89.6 ± 5	92.9 ± 2.6		
RR_sys_ [mmHg]	1	151.6 ± 28.1	147.8 ± 20.6	1.9 [− 5; 8.7]	0.659
	2	152.2 ± 15.5	150.2 ± 21.6		
RR_dia_ [mmHg]	1	92.5 ± 16.6	91.2 ± 12.8	− 0.8 [− 6.4; 4.7]	0.77
	2	94.4 ± 19.5	92.9 ± 11.5		
Heart rate [/min]	1	85.5 ± 23.2	89.1 ± 14.4	0.4 [− 6.4; 7.2]	0.90
	2	90.6 ± 12.9	88 ± 14.5		

*PaO*_*2*_ partial pressure of arterialized oxygen, *PaCO*_*2*_ partial pressure of arterialized carbon dioxide, *HCO*_*3*_^*−*^ arterialized standard hydrogen carbonate, *S*_*a*_*O*_*2*_ Oxygen saturation, *RRsys* systolic blood pressure, *RRdia* diastolic blood pressure

*Patients without long-term NIV

**Table 3:** Study II^#^: blood gas analyses and vital parameters after exercise (N = 23)

Study II^#^	Period	With pNIV	Without pNIV	Treatment effect (95% CI)	*P*
pH	1	7.37 ± 0.04	7.38 ± 0.02	**− 0.01 [− 0.02; − 0.001]**	**0.033**
	2	7.38 ± 0.03	7.38 ± 0.04		
PaCO_2_ [mmHg]	1	53 ± 2.6	46.2 ± 5.3	1.1 [− 0.04; 2.3]	0.06
	2	47 ± 4.7	51.9 ± 3.3		
PaO_2_ [mmHg]	1	55.3 ± 7	61 ± 13.4	**− 3.6 [− 6.4; − 0.8]**	**0.013**
	2	57.8 ± 9.7	59.2 ± 9.3		
HCO_3_^−^ [mmol/l]	1	29.8 ± 3.2	26.6 ± 2.1	− 0.3 [− 0.9; 0.3]	0.29
	2	26.9 ± 1.8	29.9 ± 2.6		
S_a_O_2_ [%]	1	88 ± 4.2	88.7 ± 4.7	− 0.04 [− 1.4; 1.3]	0.96
	2	89.3 ± 4.2	88.8 ± 5.3		
RR_sys_ [mmHg]	1	164.9 ± 37.2	164.8 ± 19.9	2.5 [− 8; 13]	0.63
	2	154.9 ± 13.5	160.5 ± 21.8		
RR_dia_ [mmHg]	1	95.6 ± 16.5	92.3 ± 14	1.2 [− 5.8; 8.2]	0.73
	2	89.2 ± 10.1	90.7 ± 5.8		
Heart rate [/min]	1	86.1 ± 20	97.6 ± 21.3	− 3.8 [− 8.6; 1.1]	0.12
	2	95.3 ± 20.4	91.4 ± 14.3		

Bold values represent significant results

*PaO*_*2*_ partial pressure of arterialized oxygen, *PaCO*_*2*_ partial pressure of arterialized carbon dioxide, *HCO*_*3*_^*−*^ arterialized standard hydrogen carbonate, *S*_*a*_*O*_*2*_ Oxygen saturation, *RRsys* systolic blood pressure, *RRdia* diastolic blood pressure

^#^Patients with long-term NIV

**Fig. 3:**
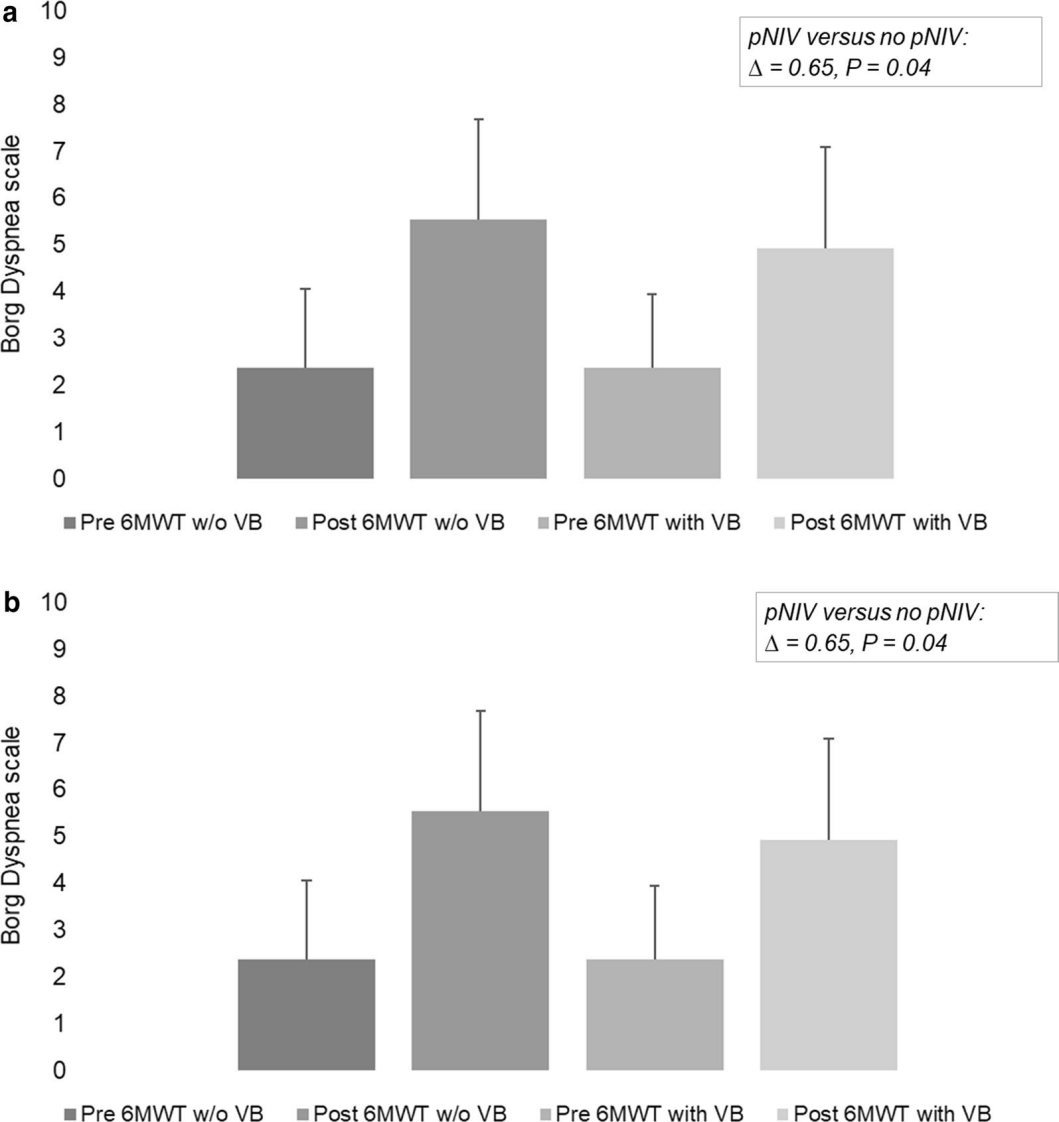
**a** Study I: Borg Dyspnea difference before and after the 6-min walking test, with and without the pNIV device (N = 38). Abreviation: *w/o* without. **b** Study II: Borg Dyspnea difference before and after 6-min walking test, with and without the pNIV device (N = 23). Abreviation: *w/o* without

**Fig. 4:**
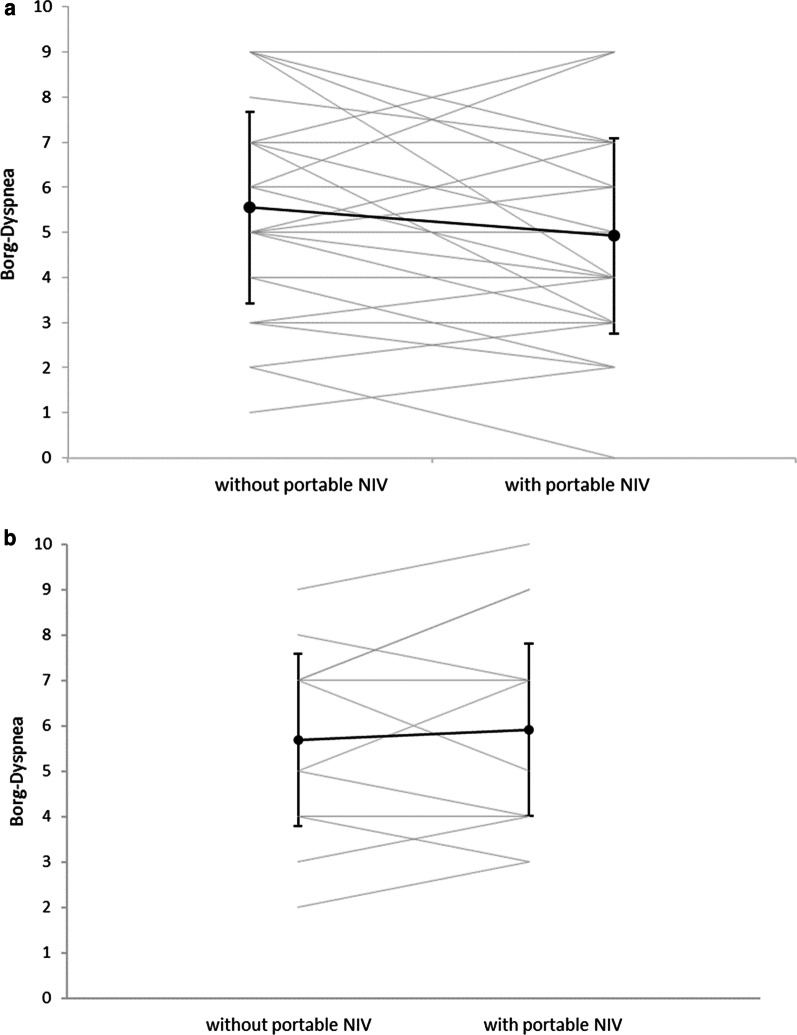
**a** Study I: The effect of individual patients on post-exercise dyspnea, with and without the pNIV device (N = 38). **b** Study II: The effect of individual patients on post-exercise dyspnea, with and without the pNIV device (N = 23)

In study I, the results of the mean BDS difference before and after exercise (primary endpoint) were 3.2 (SD ± 1.5, IQR 2–4) without pNIV compared to 2.6 (SD ± 1.8, IQR 1–4) with pNIV. This led to a mean BDS difference between the intervention and control groups of 0.65 (SD ± 1.7, p = 0.041) for Study I (Fig. 3a). Within the cohort that had already adapted to long-term NIV (Study II), the group without the pNIV showed a mean BDS difference of 4.4 (SD ± 1.85; IQR: 3–6), while the group with the pNIV had a BDS value of 4.5 (SD ± 1.9; IQR 3–6); this revealed that there were no significant differences between the two treatment interventions (− 0.09; SD ± 1.7; p = 0.55) (Fig. 3b). However, the results varied considerably amongst individual patients, with dyspnea being substantially reduced or even aggravated when using the pNIV system (Fig. 4a and b).

**Fig. 5:**
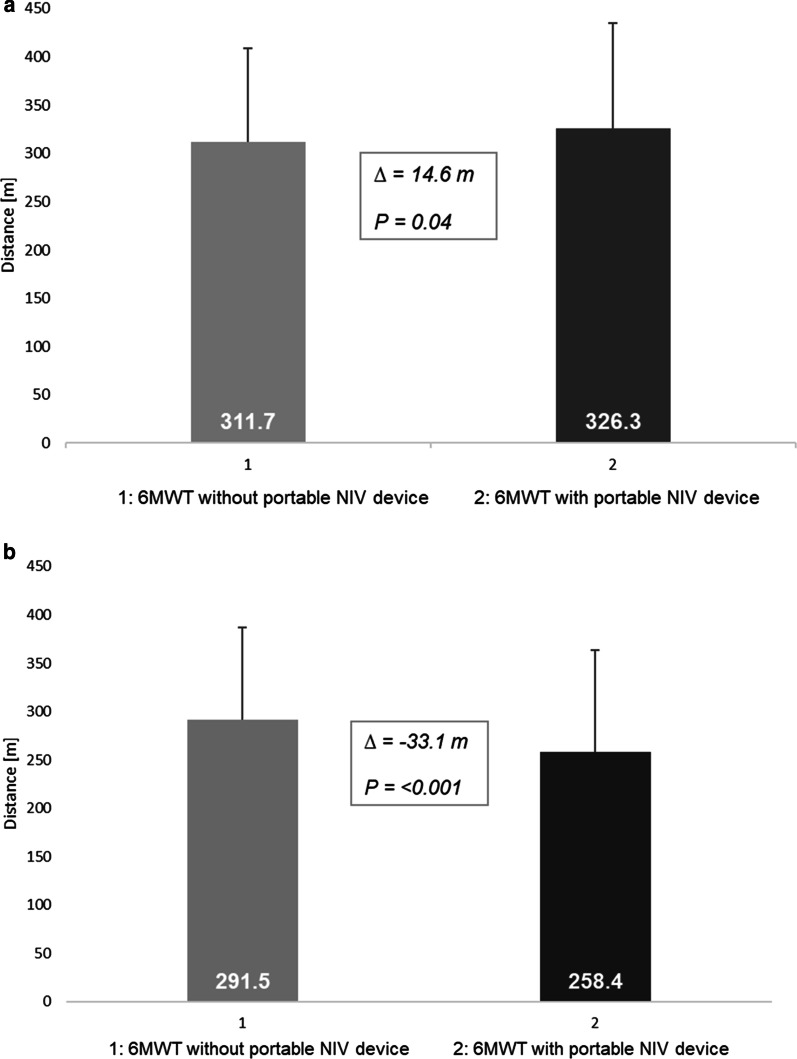
**a** Study I: Difference in distance walked during the 6-min walking test, with and without the pNIV device (N = 38). **b** Study II: Difference in distance walked during the 6-min walking test, with and without the pNIV device (N = 23)

Regarding the effect of intervention on walking distance, the use of the pNIV device led to a statistically significant improvement in the 6MWT in Study I (14.6 m; p = 0.044), but was associated with a significant deterioration in walking performance in Study II (− 33.1 m; p ≤ 0.001) (Fig. 5a, b). In Study I, 60.6% of participants reported that they benefitted subjectively from using the device, while 39.4% did not experience any benefits. Two patients who had shown a deterioration in their BDS score versus six patients with no difference in their BDS score said they had benefitted subjectively from pNIV. In a subgroup of 15 patients (Study II) who had a maximal inspiratory pressure (PImax) below 6.0 kPa, 12 of these preferred using the device while walking, while the remaining 3 did not. In a subgroup of 20 (out of 61) patients with rather low relative hyperinflation (RV/TLC < 60%), the mean difference in BDS was 3.9 without the pNIV device, and 3.8 when walking was aided by pNIV.

## Discussion

Our two randomized cross-over trials investigated the effects of a pNIV device on exercise capacity and dyspnea in COPD patients with moderate to severe airflow limitation. The major findings are as follows:

Firstly, pNIV use was found to significantly improve exercise performance in COPD patients with moderate to severe airflow limitation. This specifically pertained to improvements in both dyspnea and walking distance following a 6MWT. However, these positive effects were only observed in patients who had not become dependent on long-term NIV that had been prescribed for treating hypercapnic respiratory failure.

Secondly, in contrast to non-NIV patients, those with established long-term NIV generally did not benefit from pNIV-aided walking. Here, even walking distance and gas exchange were shown to be detrimentally affected when walking was pNIV-aided.

The current finding has several important clinical considerations. Despite reaching statistical significance, the improvements in exercise performance in the non-NIV group did not reach the internationally accepted minimal clinically important difference (MCID). Here, BDS significantly improved by 0.65 (MCID = 1) [22], while the walking distance significantly improved by 15 m (MCID = 25–35 m) [23]. Based on these findings, the use of a pNIV device to assist the patient during exercise cannot be generally recommended.

It should be noted, however, that outcome varied considerably amongst individual patients. For example, the BDS score in the non-NIV group did indeed improve by at least 1 point in 20 patients (52.6%). Substantial (ΔBDS ≥ 2) and even enormous (ΔBDS ≥ 3) improvements were seen in 12 (31.6%) and 4 patients (10.5%) in this cohort, respectively. Furthermore, even in the second group of patients who had been established on long-term NIV, clinically important improvements in BDS (ΔBDS ≥ 1) were observed in 8 patients (33.3%). Therefore, these two studies clearly show that pNIV is capable of improving exercise performance in selected COPD patients with moderate to severe airflow limitation.

The present trials also attempted to identify and define the subgroups of COPD patients that would/would not potentially benefit from pNIV use during exercise. Indeed, the current results clearly show that patients already receiving long-term NIV therapy are less likely to benefit than those who have similar lung function impairments but are not undergoing long-term NIV. This is remarkable as patients already acclimatized to positive pressures during respiration are presumed to be able to adapt more easily to pNIV. However, the opposite might also be true, since the long-term NIV patients in the current trial were already accustomed to a type of high-intensity NIV that used higher pressures than those applied during the 6MWT in the current study. Here, pNIV was restricted to 18 cmH_2_O (the fixed inspiratory pressure level provided by the device), whereas long-term NIV patients received mean inspiratory pressures of 24 cmH_2_O. The fact that inspiratory pressure levels might have been too low is also supported by recent findings in patients receiving high-intensity NIV therapy, in whom higher pressures were successfully applied during physical activity [12, 15]. Therefore, technical developments with adaptation of the pressure level in pNIV devices could potentially have an effect in patients receiving long-term NIV.

Consequently, one important limitation of the current study was that it was not possible to individually configure the settings for pNIV. Therefore, as technical developments continue to advance, there is the potential for even further improvements in exercise performance in COPD patients. Nevertheless, future technological developments need to incorporate the request to keep the weight of pNIV devices as low as possible, as holding more heavy devices is suggested to negatively impact on the treatment success especially in patients with severe COPD. Another limitation might also be anticipated in view of the rather short acclimatization period of ≥ 30 min. Since acclimatization to long-term NIV typically lasts several days, this could also be true for pNIV use during walking [24, 25].

A recent trial that used the same pNIV device as that in the current study showed that during recovery of intermittent exercise, this device is associated with a longer exercise endurance time and reduced dynamic hyperinflation (reflected by a change in inspiratory capacity and breathlessness) in patients with moderate COPD, although the MCID was also not achieved across the entire cohort [16]. A post-hoc analysis in the same trial demonstrated that patients with a lower degree of resting hyperinflation (RV/TLC: < 56 ± 2%) experienced a significant improvement when the pNIV device was used during exercise, whereas patients with more severe resting hyperinflation (RV/TLC: > 65 ± 4%) did not show improvements [26]. This could also be due to the relatively small number of sample size (n = 24) [16, 26]. We therefore tested this hypothesis in our trial, but were unable to show additional benefits in patients with reduced hyperinflation. However, these results cannot be transferred to our collective without restrictions, as the patient cohorts investigated differ significantly.

Of note, a significant number of patients in the current study showed a subjective preference towards the device, despite not having experienced benefits in BDS and walking distance. This was particularly true for patients with severely impaired inspiratory muscle strength. To this end, previous findings have shown that using a pNIV device during physical activity might help to subjectively alleviate the patient's fear of becoming short of breath [16]. This, however, requires further investigation in order to more reliably establish which COPD patient subgroups can benefit the most from pNIV-aided walking.

## Conclusion

Portable non-invasive ventilation using a hand-held device with fixed pressure settings of 18 cmH_2_O during inspiration and 8 cmH_2_O during expiration bears the potential of improving walking distance and dyspnea during exercise testing in moderate to severe COPD patients. Patients who are not already on long-term NIV do appear to benefit more from the use of pNIV than those already receiving long-term NIV therapy. Further technical developments are required, especially those aimed at allowing adjustable pressure settings. In addition, further studies are needed to better define the subgroups that would benefit the most from this adjunctive therapy.

## Data Availability

The datasets used and analyzed during the current study are available from the corresponding author on reasonable request.

## Abbreviations

6MWT: 6-Minute walking test
BDS: Borg Dyspnea Scale
BMI: Body mass index
COPD: Chronic obstructive pulmonary disease
EPAP: Expiratory positive airway pressure
FEV_1_: Forced expiratory volume in one second
FVC: Forced vital capacity
GOLD: Global initiative for obstructive lung disease
HCO_3-_: Arterialized standard hydrogen carbonate
IPAP: Inspiratory positive airway pressure
IQR: Interquartile range
LTOT: Long-term oxygen therapy
MCID: Minimal clinical important difference
NIV: Non-invasive ventilation
PaCO_2_: Partial pressure of arterialized carbon dioxide
PaO_2_: Partial pressure of arterialized oxygen
pNIV: Portable non-invasive ventilation
RR_dia_: Diastolic blood pressure
RR_sys_: Systolic blood pressure
RV: Residual volume
SaO_2_: Oxygen saturation
TLC: Total lung capacity
